# Poly(propylene 2,5-thiophenedicarboxylate) vs. Poly(propylene 2,5-furandicarboxylate): Two Examples of High Gas Barrier Bio-Based Polyesters

**DOI:** 10.3390/polym10070785

**Published:** 2018-07-17

**Authors:** Giulia Guidotti, Michelina Soccio, Nadia Lotti, Massimo Gazzano, Valentina Siracusa, Andrea Munari

**Affiliations:** 1 Department of Civil, Chemical, Environmental and Materials Engineering, University of Bologna, Via Terracini 28, 40131 Bologna, Italy; giulia.guidotti9@unibo.it (G.G.); m.soccio@unibo.it (M.S.); andrea.munari@unibo.it (A.M.); 2 Organic Synthesis and Photoreactivity Institute, ISOF-CNR, Via Gobetti 101, 40129 Bologna, Italy; massimo.gazzano@isof.cnr.it; 3 Department of Chemical Science, University of Catania, Viale A. Doria 6, 95125 Catania, Italy

**Keywords:** poly(propylene 2,5-thiophenedicarboxylate), poly(propylene 2,5-furandicarboxylate), biopolyesters, thermal properties, mechanical properties, gas barrier properties

## Abstract

Both academia and industry are currently devoting many efforts to develop high gas barrier bioplastics as substitutes of traditional fossil-based polymers. In this view, this contribution presents a new biobased aromatic polyester, i.e., poly(propylene 2,5-thiophenedicarboxylate) (PPTF), which has been compared with the furan-based counterpart (PPF). Both biopolyesters have been characterized from the molecular, thermo-mechanical and structural points of view. Gas permeability behavior has been evaluated with respect to 100% oxygen, carbon dioxide and nitrogen at 23 °C. In case of CO_2_ gas test, gas transmission rate has been also measured at different temperatures. The permeability behavior at different relative humidity has been investigated for both biopolyesters, the thiophen-containing sample demonstrating to be better than the furan-containing counterpart. PPF’s permeability behavior became worse than PPTF’s with increasing RH, due to the more polar nature of the furan ring. Both biopolyesters under study are characterized by superior gas barrier performances with respect to PEF and PET. With the simple synthetic strategy adopted, the exceptional barrier properties render these new biobased polyesters interesting alternatives in the world of green and sustainable packaging materials. The different polarity and stability of heterocyclic rings was revealed to be an efficient tool to tailor the ability of crystallization, which in turn affects mechanical and barrier performances.

## 1. Introduction

Food packaging plays a key role in the protection and preservation of all types of foods. Petrochemical plastics have been largely used as packaging material due to economical abundance and good barrier properties towards O_2_, aroma compounds, tensile strength and tear strength. Meanwhile, they have some disadvantages, the major one being non-biodegradability, which results in environmental pollution. Due to the non-renewable nature and waste disposal problem of petroleum, bioplastics have gained increasing importance in the last years. In particular, there has been an increased interest in the last 10 years from the food packaging industry towards the development and application of bioplastics for food packaging. Despite the worldwide production of bioplastics being in consistent and constant increase, there are some disadvantages that limit their use in the present time: First of all, the uneconomic feasibility of bioplastics in contrast to traditional packaging; and more importantly, the use of land for the production of bioplastics is a major hurdle in the success of bioplastic functionality. Moreover, the properties of certain bioplastics are unsatisfactory, reducing their use as films in food packaging applications. Poly(lactic acid) (PLA), for example, is characterized by thermal instability, difficult heat sealability, brittleness, low melt strength, high water vapor and oxygen permeability [1]. Other starch- and cellulose-based packaging materials due to their hydrophilic nature possess low water vapor barrier, which is responsible for poor processability, brittleness, vulnerability to degradation, limited long-term stability and poor mechanical properties [2]. In case of Poly(hydroxyalkanoate) (PHA) and poly(hydroxybutyrate (PHB), stiffness, brittleness (due to high glass transition and melting temperatures), thermal instability and poor impact resistance also restrict their applications in food packaging [3]. The aforementioned drawbacks have stimulated research into finding solutions for improving the functionality of bioplastics and developing new bioplastics.

As reported from Papageorgiou [4], vegetable feedstocks such as sugars, vegetable oil, organic acids and glycerols could be used as starting monomers for the production of new polymer. One of the most important example of monomer extracted from natural resources, such as carbohydrates and lignin, is the aromatic monomer 2,5-furan dicarboxylic acid (FDCA). The success of this biobased building block is mostly due to the synthesis of poly(ethylene 2,5-furanoate) (PEF), considered the most credible bio-based alternative to poly(ethylene terephthalate) (PET). PEF possesses superior physic/mechanical and barrier properties compared to PET [5,6,7,8]. Furthermore, recent studies have demonstrated the enzymatic degradability of PEF and PET, opening up new possibilities in eco-friendly industrial depolymerization processes for recycling purposes [9,10,11]. In particular, a recent work demonstrated the degradability of PEF by cutinase from Humicola insolens, leading to complete polymer solubilisation in 72 h of incubation [11].

For these reasons, research interest has been extended to other furan-based polyesters prepared by 2,5-furandicarboxylic acid and different glycols that showed very smart barrier properties [12,13].

This year, we proposed a novel homopolymer with outstanding gas barrier properties as *alter egos* of FDCA-based polyesters, i.e., poly(butylene 2,5-thiophenedicarboxylate) (PBTF) [14,15]. Both starting materials, 2,5-thiophenedicarboxylic acid (TFDCA) and 1,4-butanediol (BD), can be derived from renewable resources, thus obtaining a fully biobased polymer. TFDCA is indeed industrially produced from the reaction of adipic acid with thionyl chloride [16,17]. In turn, adipic acid can be obtained from glucaric or muconic acid [18]. 

In light of the scenario described above, in the present contribution, poly(propylene 2,5-thiophendicarboxylate) (PPTF) has been synthesized for the first time, and characterized from the molecular, thermal, diffractometric, mechanical and barrier behavior points of view. Poly(propylene 2,5-furandicarboxylate) (PPF) has been also prepared in order to evaluate the effect of different heterocyclic five-membered aromatic ring on the final biopolyester properties. Both polyesters under study are 100% biobased, the 1,3-propanediol being obtainable from bioconversion of glycerol [19].

To the best of our knowledge, no studies regarding the effect of substitution of the furan ring with thiophene have been described in the literature.

## 2. Materials and Methods 

### 2.1. Materials

TFDCA (97%) was purchased from TCI (Tokyo, Japan), whereas FDCA (98%) from CHEMOS GmbH & Co. K (Regenstauf, Germany). 1,3-propanediol (1,3-PD), hexafluoro-2-propanol, chloroform, methanol, BD (99%), titanium tetrabutoxide (TBT, 97%) and titanium isopropoxide (TTIP, 97%) were obtained from Sigma Aldrich (St. Louis, MO, USA). TBT and TTIP were both distilled before use, while other products were used as received.

### 2.2. Polyester Synthesis

PPTF was prepared by two-step melt polycondensation by reacting TFDCA (15.2 g, 0.088 moL) with PD (19.9 g, 0.262 moL) in the presence of 200 ppm/g_polymer_ of TBT and 200 ppm/g_polymer_ of TTIP. A 200 mL glass reactor was placed in a silicon oil bath and the reaction mixture was stirred at 100 rpm by a two-bladed centrifugal stirrer connected to an overhead motor (IKA-Werke GmbH & Co., Staufen, Germany). Nitrogen flow was applied and the temperature was set to 170 °C. When more than 90% of the water produced during esterification was distilled off (about 3 h), pressure and temperature were gradually reduced to 0.1 mbar and increased to 200 °C, respectively. Polymerization was stopped when constant torque was measured (4 additional hours). As far as PPF is concerned, a molar diacid/glycol ratio of 1:7 and antimony trioxide (10.6 mg per gram of final theoretical polymer) were employed. The glycol was used in large excess in order to enhance FDCA solubilization. All the reagents and catalysts were loaded into the polymerization reactor simultaneously and constantly mixed by a mechanical stirrer. After around 30 min, the mixture turned out transparent indicating the solubilization of the acid in the glycol. For the subsequent two hours, the temperature was fixed at 180 °C under controlled nitrogen flow in presence of a reflux condenser, to limit the leakage of BD from the reaction medium. Then, the temperature was raised at 200 °C and the reflux condenser taken off (1 h and half). In the second stage, the temperature was increased until 220 °C, while the pressure was gradually reduced to 0.1 mBar. This stage was stopped when the torque reached a constant value (2 h and half).

The as-synthesized polymers were purified through dissolution in a mixture hexafluoro-2-propanol/chloroform and precipitation in methanol. The purified polymers, in the form of white floccules, were dried at 30 °C under vacuum to constant weight. Thin films of about 150 µm of thickness, were obtained by compression molding using a Carver press. Purified polymer was melted 30 °C above their melting temperature and kept for 2 min at a pressure of 5 tons/m^2^. Lastly, films were cooled to RT in press by tap water.

Film thickness was determined by Sample Thickness Tester DM-G (Brugger Feinmechanik GmbH, Munich, Germany). Reported value represents the mean thickness of three experimental tests, each run on 10 different points of the polymer film surface at RT.

### 2.3. Molecular, Thermal and Structural Characterization

Polymer structure was checked by ^1^H-NMR spectroscopy at RT. A Varian Inova 400-MHz (Palo Alto, CA, USA) was used for the measurements.

Molecular weight was determined by gel-permeation chromatography (GPC) at 30 °C with a 1100 HPLC system equipped with PLgel 5-μm MiniMIX-C column ((Santa Clara, CA, USA). A UV-detector (Santa Clara, CA, USA) was employed. A Hexafluoro-2-propanol/chloroform mixture (5:95 *v*/*v*) was used as eluent with a 0.3 mL/min flow. A molecular weight calibration curve was obtained with polystyrene standards in the molecular weight range 800–100,000 g/moL.

TGA was carried out under nitrogen atmosphere by means of a Perkin Elmer TGA7 apparatus (Waltham, MA, USA). Gas flow of 30 mL/min and heating scan of 10 °C/min were used for the analysis.

A Perkin Elmer DSC6 (Waltham, MA, USA) was used for the calorimetric measurements. Weighed samples were encapsulated in aluminum pans and heated to about 40 °C above fusion temperature at a rate of 20 °C/min (first scan), held there for 3 min, and then quenched to −40 °C. Finally, they were reheated from −10 °C to a temperature well above the melting at a heating rate of 20 °C/min (second scan). 

X-ray diffraction (XRD) patterns of polymeric films were carried out by using a PANalytical X’PertPro diffractometer (Almelo, The Netherlands) equipped with a copper anode (λ = 0.15418 nm) and a fast solid state X’Celerator detector (Almelo, The Netherlands). Data were collected in the 3–60° 2θ interval (collecting time 100 sec/step, 0.10 °/step). The indices of crystallinity (χ_c_) were evaluated from the XRD profiles by the ratio between the crystalline diffraction area (A_c_) and the total area of the diffraction profile (A_t_), χ_c_ = A_c_ × 100/A_t_. The crystalline diffraction area has been obtained from the total area of the diffraction profile by subtracting the amorphous halo. The amorphous was modeled as bell shaped peak baseline. The non-coherent scattering was previously subtracted.

The in situ XRD patterns were collected by means of an Anton Paar (Graz, Austria) TTK450 hot stage mounted inside the diffractometer (5–40° 2θ interval; collecting time 40 sec/step, 0.10°/step).

### 2.4. Mechanical Properties 

Tensile measurements were carried out on rectangular films (5 mm wide and 0.2 mm thick) with a crosshead speed of 10 mm/min by using a Instron 4465 tensile testing machine (Norwood, MA, USA), equipped with a rubber grip and a 100 N load cell. A preload of 1 MPa was applied to each specimen prior to testing. At least five replicates were run and the results are provided as the average ± standard deviation.

The film thickness was determined using a Digital Dial Indicator (MarCator 1086 type, Mahr GmbH, Esslingen, Germany), connected to a PC, using the Sample Thickness Tester DM-G software. The reading was made measuring a minimum, a maximum and an average value. The thickness value is expressed in micron, with a resolution of 0.001 μm. The reported results represent the mean value thickness of three experimental tests run at 10 different points on the polymer film surface at room temperature. The film thickness was approximately of 200 and 120 micron for PPF and PPTF, respectively.

### 2.5. Water Contact Angle Measurements

Static contact angle measurements were performed on polymer films by using a KSV CAM101 instrument (KSV Instruments, Helsinki, Finland) by recording the side profiles of deionized water drops for image analysis. Eight drops were observed on different areas for each film, and water contact angles (WCA) were reported as the average value standard deviation.

### 2.6. Gas Transport Measurements

The determination of the gas barrier behavior was performed by a manometric method using a Permeance Testing Device, type GDP-C (Brugger Feinmechanik, GmbH, München, Germany), according to ASTM 1434-82 (Standard test Method for Determining Gas Permeability Characteristics of Plastic Film and Sheeting), DIN 53 536 in compliance with ISO/DIS 15 105-1 and according to *Gas Permeability Testing Manual* (Registergericht München HRB 77020, Brugger Feinmechanik GmbH). 

After a preliminary high vacuum desorption of the lower analysis chamber, the upper chamber was filled with the gas test, at ambient pressure. A pressure transducer, set in the lower chamber, records continuously the increasing of gas pressure as a function of the time. The gas transmission rate (GTR, expressed in cm^3^·m^−2^·day^−1^·bar^−1^) was determined considering the increase in pressure in relation to the time and the volume of the device. All measurements have been carried out at room temperature of 23 °C. The operative conditions were: Gas stream of 100 cm^3^·min^−1^; 0% RH of gas test, food grade; sample area of 78.5 cm^2^ (standard measurement area). Films were analyzed at a temperature of 8, 15, 23 and 38 °C. Gas transmission measurements were performed at least in triplicate and the mean value is presented. Method A was used for the analysis, as just reported in the literature [20,21], with evacuation of up/lower chambers. Sample temperature was sets by an external thermostat HAAKE-Circulator DC10-K15 type (Thermo Fischer Scientific, Waltham, MA, USA). 

The transport phenomena background followed in the experiment is well described in literature, with a full description of the mathematical equation and interpretation [22].

### 2.7. Relative Humidity

According to the procedure reported on the *Gas Permeability Testing Manual* [21], the analyses were performed at two relative humidities (RH) obtained with saturated saline solutions:

- Standard Atmosphere, 23 °C, 85% of RH, with saturated KCl solution;
- Tropical Climate, 38 °C, 90% RH, with saturated KNO_3_ solution, according to the DIN 53 122 part 2 norm.

A round paper filter (Macherey-Nagel 85/70 BF 70 mm diameter, Düren, Germany) was inserted in the humid part of the top permeation cell, humidified with the desired saturated saline solution. Method C was used, with gas flow blocked onto the test specimen during evacuation. In this manner, the test gas was humidified inside the permeation cell. This method evacuates only the area of the bottom part of the sample while on the top part of the test specimen, with the humidified gas, the normal ambient pressure was applied.

## 3. Results and Discussion

### 3.1. Molecular Characterization

**Figure 1:**
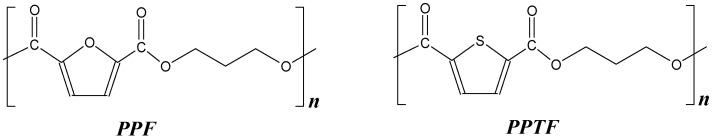
Poly(propylene 2,5-furandicarboxylate) (PPF) and poly(propylene 2,5-thiophendicarboxylate) (PPTF) chemical structure.

The as-prepared samples appeared as yellowish hard solid materials; the purified ones were white floccules. Their chemical structures are shown in Figure 1: As can be seen, the two homopolymers display a similar chemical structure, being both aromatic polyesters with same glycol sub-unit, although differing for the aromatic acid sub-unit, containing a furan ring in the case of PPF, and a thiophene one in PPTF. 

**Figure 2:**
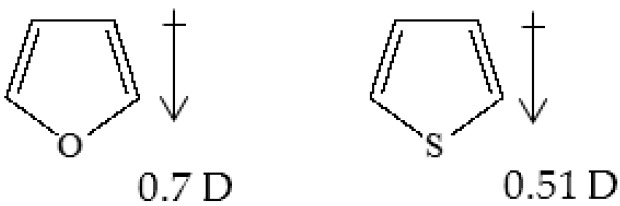
Dipole moment vector of furan and thiophene rings.

The two heteroaromatic five-membered rings are characterized by some differences, first of all, the resonance energy has a different value: 6.2 Kcal/mole for furan and 29.1 Kcal/mole for thiophene, respectively. The resonance stabilization of heteroaromatic molecules depends on several factors, which arise from the atomic features of the heteroatom and result in different molecular properties. The ability of one of the heteroatom’s non-bonding electron pairs to participate in the aromatic π-system is critical. Oxygen, being highly electronegative, shares its non-bonding electron pair less willingly than sulfur. Sulfurs’ lower electronegativity, a result of its larger size and hence softness, permits greater participation of its non-bonding electron pair to the aromatic π-system. Therefore, the furan ring is the least aromatic, because of the highest electronegativity of oxygen. Analogously, the electronegative character of the heteroatoms determines a dipolar moment on the ring, the negative pole being located at the heteroatom. Once again, the dipole moments of furan and thiophene are different and function of the heteroatom electronegativity: The higher the electronegativity, the higher the dipole moment (Figure 2). 

**Figure 3:**
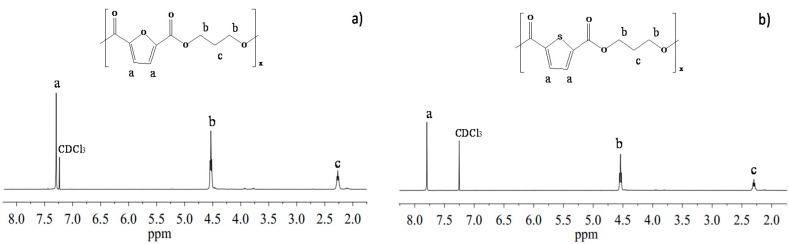
^1^H-NMR spectrum of: (**a**) PPF; (**b**) PPTF with resonance assignments.

As far as the two polyesters under investigation are concerned, ^1^H-NMR analysis confirmed the expected structure (Figure 3) with no impurities in the spectrum. Both polymers are characterized by high and similar molecular weight and by same narrow polydispersity index (Table 1), proving that optimized reaction conditions were achieved.

It is worth highlighting the reagents were used as received and the adopted protocol is very close to industrial procedures for the preparation of polyesters. The synthesized polyesters were filmed by compression molding and rapid cooled in ice water.

As shown by the WCA data reported in Table 1, PPF film appeared to be more hydrophilic than the PPTF one, in agreement with its higher dipole moment.

### 3.2. Thermal Characterization

**Figure 4:**
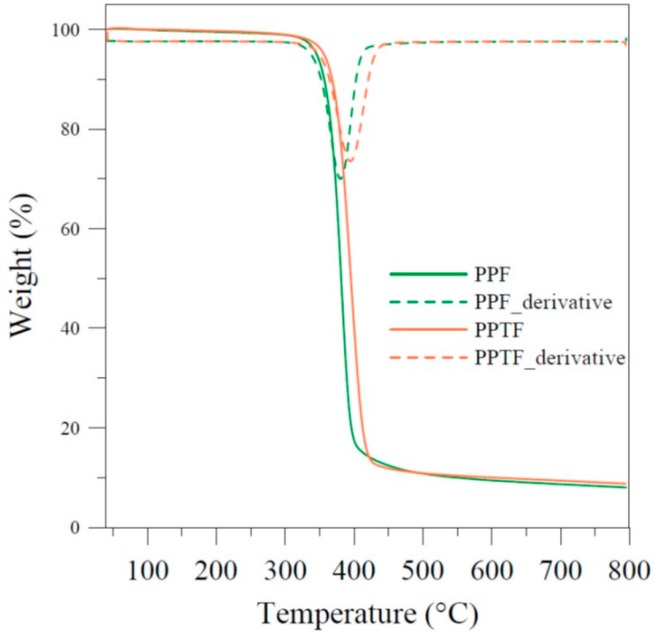
Thermogravimetric curve (solid line) and corresponding derivative (dashed line) under nitrogen flow for PPF and PPTF.

Afterwards PPF and PPTF samples have been subjected to thermogravimetric analysis (TGA) under nitrogen flux. The temperatures relative to the degradation onset T_onset_ and to the maximum weight loss rate T_max_ have been reported in Table 1, whereas the TGA curves together with the corresponding derivatives are shown in Figure 4: Both polyesters displayed good thermal stability, the degradation process occurring in one step, with a residual mass of 6% at 800 °C. Anyway, PPTF appeared to be more thermally stable than PPF. This result can be ascribed as due to: (i) the higher resonance energy, (ii) p to d π-back bonding and (iii) lack of ring strain because of the longer C–S bond for PPTF.

The main thermal transition data of both powder samples (labeled as PPF_P and PPTF_P) and polymer films (labeled as PPF_F and PPTF_F) are reported in Table 1.

**Figure 5:**
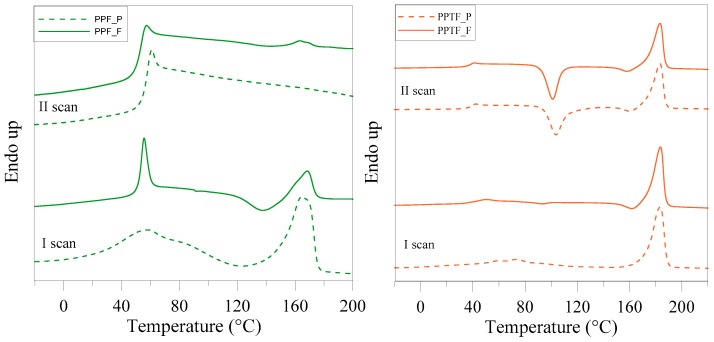
Calorimetric traces of PPF and PPTF powder and film (20 °C/min): 1st scan and 2nd scan after melt quenching.

From the data reported in Table 1 and from the DSC curves of Figure 5, one can see that the two homopolymers in forms of powder displayed identical phase behavior: both were indeed semicrystalline samples, even though PPF was able to crystallize during heating scan once T_g_ is exceeded (∆H_cc_ < ∆H_m_).

The higher crystallinity degree of PPTF indicates a higher crystallizing ability with respect to PPF.

As to the glass transition phenomenon, both T_g_ values are above room temperature, indicating that in both polymers a glassy amorphous phase is present. T_g,PPF_ > T_g,PPTF_ despite the higher crystallinity degree of PPTF, due to stronger interchain interactions present in PPF because of higher electronegativity of oxygen atoms. The T_g_s of the powders are higher than the corresponding films, as expected considering their higher crystallinity degree, due to solvent induced crystallization.

The higher melting temperature of PPTF can be associated with a crystalline phase characterized by a higher degree of perfection, due to both the higher mobility of macromolecular chains and the higher aromaticity of thiophene rings, which favors the chain folding.

As a matter of fact after melt quenching, the two polyesters, independent of whether they are in film or powder form, are characterized by a different phase behavior: PPF is completely amorphous, as evidenced by the corresponding DSC trace exclusively showing the baseline deviation associated to the glass transition phenomenon, whereas PPTF is semicrystalline. In this last case, the DSC trace evidenced the glass transition phenomenon followed by a cold crystallization peak and a melting peak at higher temperature. The heat of crystallization is however lower than the heat of fusion indicating that the material cannot be vetrified in a completely amorphous state by quenching. The behavior after melt quenching confirmed the higher crystallizing ability of PPTF with respect to PPF. The T_g_ values of 2nd scan are in line with those of the 1st scan, the T_g_ of PPTF being lower than that of PPF.

### 3.3. Diffractometric Characterization

**Figure 6:**
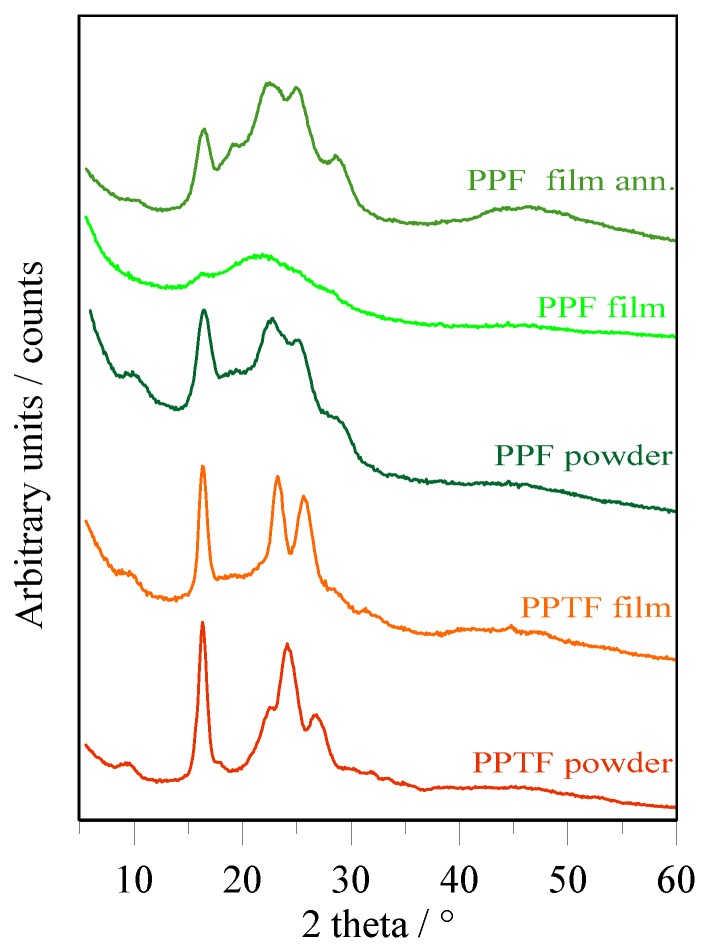
X-ray diffraction profiles of PPTF powder, PPTF film, PPF powder, PPF film and PPF film after annealing for 45 min. at 110 °C.

X-ray diffraction (XRD) measurements were carried out to investigate the nature of the crystal phase. Collected patterns are displayed in Figure 6. PPTF powder sample showed most significant peaks at 2θ values of 9.2°, 16.3°, 17.9°, 22.5°, 24.1°, 26.6° (*d* = 9.6, 5.4, 4.9, 3.9, 3.7, 3.3 Å) and crystallinity index X_c_ = 46 (±3)%. PPTF film was characterized by reflections at 2θ of 9.3°, 16.3°, 23.3°, 25.6° (*d* = 9.5, 5.4, 3.8, 3.5 Å) and X_c_ = 43 (±3)%. The peak positions and shape of the two patterns were different enough to assign them to different crystal phases, hereinafter named α-PPTF in case of powder sample, and β-PPTF for film sample.

As shown in Figure 6, PPF film after preparation shows a very poor crystallinity (X_c_ = 3 (±2) %), but the thermal treatment at 110 °C for 45 min. provoked a significant increase in crystallinity (X_c_ = 31 (±3)%). It became very similar to the one of powder sample (X_c_ = 37 (±3)%). The XRD patterns of PPF, either in powder or film after annealing, were similar and can be attributed to the same (a unique) crystal phase. This had main reflections at 10.2°, 16.4°, 19.1°, 22.6°, 25.0°, 28.6° (corresponding to *d =* 8.7, 5.4, 4.6, 3.9, 3.6, 3.1 Å). XRD analysis evidenced that the PPF sample is characterized by a lower degree of crystallinity, in perfect agreement with calorimetric results.

**Figure 7:**
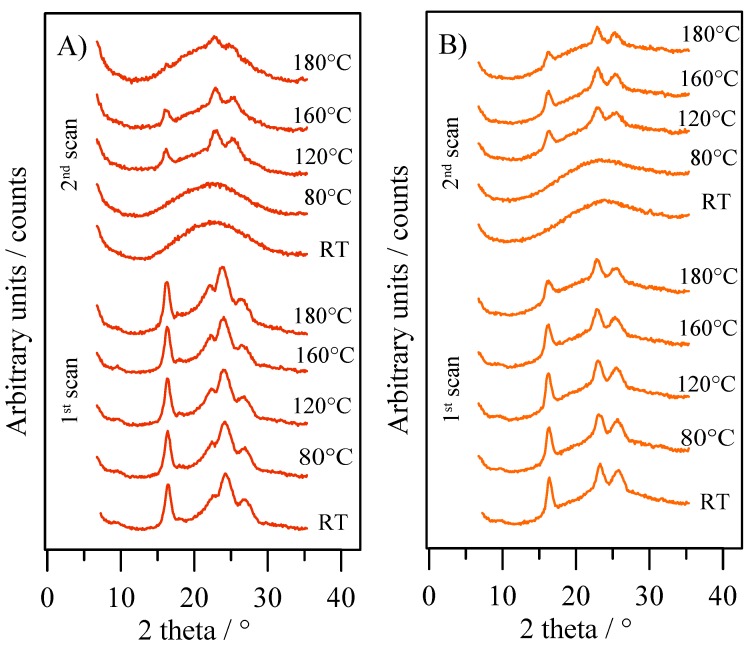
XRD patterns collected in situ at the indicated temperatures for PPTF powder (**A**) and PPTF film (**B**).

The thermal stability of the two different crystal phases present in PPTF powder and film samples were checked by in situ XRD measurements (see Figure 7). During the 1st scan process both samples showed their own crystal phase up to melting, no transformation of a phase in the other by solid-solid transition being appreciated. On the contrary, as pointed out by the patterns collected during 2nd scan, after melt quenching, the powder sample also crystallized according to the β-form, suggesting that β crystal phase could be the more stable one.

### 3.4. Mechanical Characterization

The results of tensile tests are reported in Table 1. The slightly higher elastic modulus of PPTF can be explained on the basis of the crystalline phase present in this sample; the stress at break of PPF is, on the contrary, 2.5 times higher than that of PPTF because of its higher T_g_ value. Lastly, the elongation at break for the two samples was very similar. No significant effect of kind of ring on mechanical properties was thus evidenced. Moreover, the two polyesters showed similar characteristics, i.e., very high elastic modulus and brittle fracture, to that of PEF.

### 3.5. Gas Permeability Characterization

The barrier properties have been evaluated on respect three different dry gases, normally used for food packaging application, i.e., N_2_, O_2_ and CO_2_. The GTR values normalized for the sample thickness and measured at 23 °C and at 0% of humidity are collected in Table 1: both polymers under study are characterized by outstanding barrier performances, comparable to that of PBTF [14] and superior to PEF ones [6,7]. 

The outstanding barrier properties of furan-based polymers are also supported by structural and dynamical studies showing the hindering of ring flipping [5] and the limited subglass local dynamics [23] imparted by furanic moiety. In addition, as suggested by Araujo et al., the ability of stopping the gas passage is also due to the establishment of C–H∙∙∙O interactions among adjacent polymer chain segments. The authors also state that the amount of intermolecular C−H···O contacts is particularly favored when FDCA subunits are in the syn conformation, as in the crystalline PEF lattice [24]. The syn/anti conformations ratio also depends on the glycol methylene group number. We can hypothesize that the presence of an additional CH_2_ group in the PPF glycol subunit with respect to PEF, can favor the syn conformation of FDCA and consequently increase the C–H∙∙∙O interactions, improving the barrier properties of PPF.

Moreover, as can be evicted from the experimental data, for both the samples under study, CO_2_ is more permeable than O_2_ and N_2_, as observed for other similar polymers previously investigated [25,26], due to diffusivity drop and solubility increment with decreasing permeant size (molecular diameter of CO_2_ 3.4 Å, oxygen molecular diameter 3.1 Å and nitrogen molecule diameter 2.0 Å, respectively) [27]. However, it is worth noting that the gas transmission rates to oxygen and carbon dioxide were not so different, analogously to PEF polyester previously investigated [7]. The polar character of furan and thiophene moieties determined a high affinity (high solubility) of CO_2_ gas molecules with polymer matrix.

Barrier properties with respect to PPF: This result can be explained considering that PPTF film is semicrystalline while PPF one is amorphous. As is well known from literature [28], several factors could influence the final barrier behavior of the materials, such as crystallinity, chain polarity, chain flexibility, molecular weight and distribution of molecular weight. In particular, the crystallinity plays a key role since the gases cannot diffuse and permeate in the crystalline phase, due to the restricted polymer chain mobility in this phase. In general, polymer crystals are impenetrable to gas molecules and consequently the polymers with the higher percentage of crystalline phase are the best barrier materials.

#### 3.5.1. Activation Energy

As is well known, permeability depends on temperature according to an Arrhenius-type equation, well described in literature [20,29]. Gas transmission rate increases exponentially with the temperature and the activation energy (E_a_) is a measure of the energy required to start the permeation process. By plotting the LnGTR against the reciprocal temperature 1/T expressed in K, a linear curve is obtained. The activation energy is deduced by calculating the value of the slope (−E_a_/R) of the Arrhenius straight line, where R is the gas constant (8.314 J/moL K).

We evaluated the activation energy for CO_2_ gas test: Values of 32 KJ/moL and of 20 KJ/moL were determined for PPF and PPTF, respectively (with R^2^ = 0.9, for both samples), in good agreement with the value found by Burgess et al. for PEF (23.7 KJ/moL) [7]. The higher activation energy found for PPF can be correlated to the higher polarity of the furan ring compared to the thiophene one, which determines a higher affinity of carbon dioxide molecule for PPF.

#### 3.5.2. Influence of Relative Humidity on the Gas Transmission Rate

**Figure 8:**
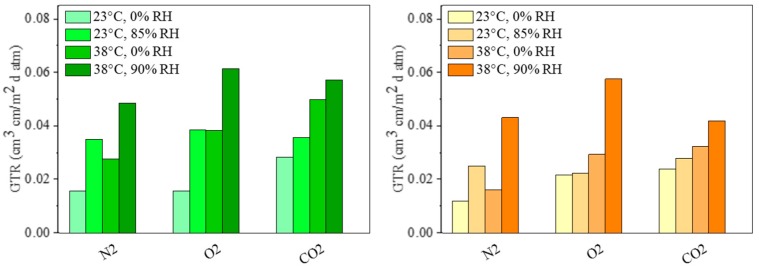
GTR at 23 °C and 38 °C 0% RH, at 23 °C and 85% RH, at 38 °C and 90% RH.

In order to explore a future application of PPTF and PPF as packaging materials, the effect of RH and temperature on the gas transmission was investigated. Figure 8 visualizes the measured values of the GTR with RH gradients of 85% at 23 °C and of 90% at 38 °C respectively. These conditions were chosen to describe the standard atmosphere and tropical ambient, respectively, as reported on the *Gas Permeability Testing Manual* [21].

The results showed that the GTR values of both PPF and PPTF films increased with increasing RH, in agreement with data described in literature for other polymers [29,30,31]. The water vapor molecules that goes through the polymer membrane can acts as plasticizer, giving rice to an increase in polymer free volume, with consequent higher gas permeability [30]. The effect is particularly evident in the case of nitrogen and oxygen, while in contrast being more modest for carbon dioxide. This result could be due to the higher affinity (solubility) of CO_2_ with both polymer matrices. It has to be reminded that furan rings, as well as thiophene ones, are polar moiety and carbon dioxide molecule is characterized by an induced dipole moment. 

Table 2 collects the increment, expressed as in percentage terms, in respect to the value determined at 23 and 38 °C, respectively. At 23 °C, the major increments of GTR were recorded for PPF sample: this trend could be explained as due to the higher plasticizer effect of water in case of PPF. PPF macromolecular chains are indeed characterized by a higher polarity, in comparison to the ones of PPTF, as proved by the higher dipole moment associated to furan ring.

Consequently, PPTF film showed excellent moisture resistance.

An opposite result was obtained at 38 °C: this temperature is very close to PPTF glass transition temperature. At this temperature, macromolecular cooperative motions start with a significant raise of free volume, and hence of frequency of successful gas diffusive jumps. 

## 4. Conclusions

Two 100% biobased aromatic polyesters were successfully synthesized by two-stage melt polycondensation, a solvent-free synthetic approach easily scaled up at industrial level. They differed in the diacid sub-unit, with the one of PPF containing the furan ring, in which the PPTF is replaced with thiophene. The thiophene ring is characterized by higher resonance energy, and lower dipole moment compared to the furan ring, which impacts on the solid-state properties: PPTF is indeed more thermally stable, its T_g_ is lower, whereas the crystallizing ability is higher with respect to PPF. The different characteristics of the two ethero-aromatic five membered rings reflect on the barrier properties, which were outstanding for both biopolyesters, being even superior to PEF. Nevertheless, the measurements performed at different RH showed that the gas transmission increased with increasing RH, the increment being more modest for PPTF at 23 °C, due to a lower plasticizer effect of water because of the lower polarity of thiophene ring compared to furan one. An opposite result was obtained at 38 °C, with PPF appearing more performant than PPTF. The temperature of 38 °C, which mimics tropical climate, is very close to PPTF’s T_g_ and therefore is where a significant increment of free volume occurs.

In conclusion, both biopolyesters here presented represent excellent candidates for sustainable packaging applications, PPTF being preferable in areas with a temperate climate, and PPF in tropical regions.

## Figures and Tables

**Table 1:** Molecular, thermal and mechanical characterization data for PPF and PPTF.

	**PPF**		**PPTF**	
**MOLECULAR CHARACTERIZATION**				
M_n_ (g/moL)	30,000		26,300	
D	2.3		2.3	
WCA (°)	90 ± 3		94 ± 3	
**THERMAL CHARACTERIZATION**				
**Thermogravimetric analysis**				
T_onset_ (°C)	360		373	
T_max_ (°C)	387		396	
**Differential scanning calorimetry**				
**1st scan**				
	**powder**	**film**	**powder**	**film**
T_m_ (°C)	165	169	183	184
ΔH_m_ (J/g)	26	7	39	37
T_g_ (°C)	56	52	45	40
ΔC_p_ (J/g°C)	0.278	0.361	0.128	0.229
T_c_ (°C)	119	138	-	161
ΔH_cc_ (J/g)	7	7	-	3
**2st scan**				
	***powder***	**film**	**powder**	**film**
T_m_ (°C)	-	-	183	183
ΔH_m_ (J/g)	-	-	34	36
T_g_ (°C)	52	52	39	38
ΔC_p_ (J/g°C)	0.361	0.359	0.315	0.227
T_c_ (°C)	-	-	104	101
ΔH_cc_ (J/g)	-	-	30	31
**MECHANICAL CHARACTERIZATION**				
E (MPa)	1363 ± 158		1419 ± 165	
σ_B_ (MPa)	31 ± 3		12 ± 4	
ε_B_ (%)	3 ± 1		2 ± 0.5	
**GAS PERMEABILITY CHARACTERIZATION AT 23 °C RH 0%**				
O_2_-GTR	0.0224 ± 3 × 10^−6^		0.0202 ± 14 × 10^−5^	
CO_2_-GTR	0.0288 ± 3 × 10^−6^		0.0243 ± 13 × 10^−5^	
N_2_-GTR	0.0157 ± 6 × 10^−5^		0.0120 ± 2 × 10^−5^	

**Table 2:** Percent of GTR increment at 23 °C and 85% of RH and at 38 °C and 90% of RH.

Gas	PPF		PPTF	
	23 °C, 85% RH	38 °C, 90% RH	23 °C, 85% RH	38 °C, 90% RH
N_2_	+123	+75	+109	+167
O_2_	+77	+60	+3	+96
CO_2_	+23	+15	+17	+30
